# Enhancing Cutting Rates in Multi-Channel HSWEDM of Metal Materials with a Novel Decoupling Circuit

**DOI:** 10.3390/mi14122226

**Published:** 2023-12-11

**Authors:** Guokang Su, Chuanyun Zhang, Junfei Li, Guixian Liu, Xiaolei Chen, Yongjun Zhang

**Affiliations:** 1 State Key Laboratory of Precision Electronic Manufacturing Technology and Equipment, Guangdong University of Technology, Guangzhou 510006, China; su_guokang@163.com (G.S.); jfli17818588110@163.com (J.L.); gxliu@gdut.edu.cn (G.L.); 2 Guangzhou Key Laboratory of Nontraditional Machining Technology and Equipment, Guangdong University of Technology, Guangzhou 510006, China; 3 School of Mechanical Engineering, Xi’an Technological University, Xi’an 710021, China; cyzhang@xatu.edu.cn

**Keywords:** multi-channel HSWEDM, metal workpieces, electrical signal coupling, decoupling circuit, synchronous discharge, cutting rate

## Abstract

Multi-channel high-speed wire electrical discharge machining (HSWEDM) has shown great potential in enhancing the cutting rate of metal workpieces. However, the mechanism of multi-channel discharges in this technique remains unclear. In this paper, the equivalent circuit and processing model of the multi-channel HSWEDM were developed to investigate the discharge characteristics. It was found that the equipotential between electrodes is the primary factor causing electrical signal coupling between channels, hindering the achievement of synchronous discharge. To address this issue, a novel power supply with a decoupling circuit was devised. By utilizing the combined effect of electrode wire resistance and current limiting resistance (*R_c_*), a potential difference was induced between electrodes in different channels, enabling electrical signal decoupling and facilitating synchronous discharge. The impact of *R_c_* on synchronous discharge was examined, revealing that a reduction in *R_c_* can increase the gap voltage of non-breakdown channels, thereby enhancing the discharge ratio. Finally, cutting rate experiments were conducted. When the new power supply was used for electrical signal decoupling, the cutting rates of multi-channel WEDM were significantly improved. Compared to single-channel HSWEDM, the cutting rates of two-channel and four-channel HSWEDM are enhanced by 84.06% and 247.83%, respectively.

## 1. Introduction

Wire electrical discharge machining (WEDM) is a non-contact machining technique that utilizes thermal erosion caused by spark discharges to process workpieces. Based on the wire running system, WEDM can be classified into two technologies: low-speed WEDM (LSWEDM) and high-speed WEDM (HSWEDM). Due to its ability to process workpieces of any hardness and its high material utilization efficiency, WEDM has become an indispensable technology in industries such as mold manufacturing [1], automobile manufacture [2], biomedicines [3] and aerospace [4]. In some industries, large annular metal workpieces need to be cut into multiple parts according to design specifications, and WEDM is the primary method employed. However, the lengthy cutting cycle has been problematic for enterprises, and increasing the cutting rate of WEDM is the most direct solution. Scholars have explored mechanisms analysis [5], parameter optimization [6], intelligent control [7], and auxiliary processes [8], which have significantly improved the cutting rate of WEDM, although they are approaching their limits.

**Figure 1:**
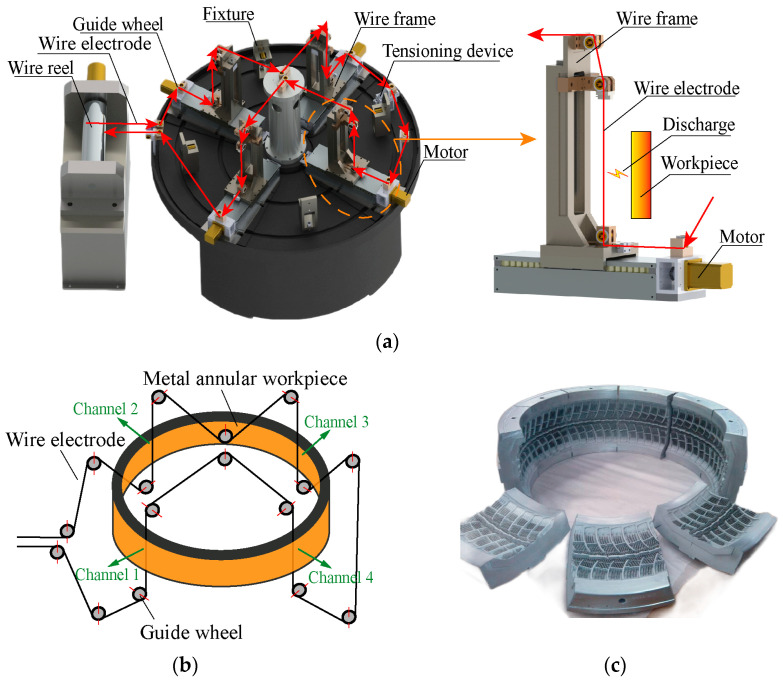
Multi(four)-channel machine tool: (**a**) 3D drawing of multi-channel machine tool; (**b**) wire running path; (**c**) tire mold after being cut.

In order to enhance the cutting rate of annular metal workpieces, a novel multi-channel HSWEDM machine tool has been proposed. This machine tool is capable of cutting workpieces across different channels using only one wire electrode. Figure 1a illustrates the multi-channel machine tool, where the wire electrode is guided by specialized guide wheels on a circular worktable and sequentially passes through wire frames at different channels. At each channel, the electrode wire is driven by an independently controlled wire frame to perform radial cutting from the inside out on the annular workpiece. The wire running path of the multi-channel machine tool is depicted in Figure 1b. Additionally, Figure 1c shows a tire mold that has been divided into several parts to meet the requirements of demolding. Theoretically, this innovative machine tool holds the potential to significantly increase the cutting rate. However, utilizing a single wire electrode to process a single metal workpiece across different channels leads to electrical signal coupling between the channels, involving a multi-channel discharge problem and posing challenges in accurately identifying the discharge status of each channel and implementing effective servo control for achieving the desired high cutting rate. This phenomenon highlights a long-standing issue in multi-channel discharge machining of metal workpieces, necessitating further investigation into the underlying mechanisms and the development of efficient technical solutions to overcome these challenges.

The multi-channel machine tool is a new type of machine tool. Although there has been no research directly related to this technique, a similar multi-channel WEDM approach for semiconductor materials have been widely discussed. This approach uses an electrode wire that is repeatedly wound between a pair of multi-groove regulating wheels to form a number of parallel threads. The wire is then fed in the same direction to allow the multi-wire cutting of workpiece [9]. Itokazu et al. successfully developed the 10-channel WEDM technology to process silicon carbide blocks at a speed of 56 µm/min [10], and the 40-channel WEDM technology at a speed of 80 µm/min [11]. Okamoto [9] employed a wire electrode with a track-shaped section in multi-channel WEDM to enhance the operational stability of the electrode wire and improve the quality of the kerf shape of SiC. Multi-channel WEDM can significantly improve the cutting rate in the processing of semiconductor materials through multi-channel synchronous discharge. Chen et al. [12] elucidated the mechanism of multi-channel synchronous discharge and identified the body resistivity of semiconductor materials as a crucial factor in achieving multi-channel discharge processing. However, due to the high conductivity and negligible body resistivity of metal workpieces, achieving synchronous discharge at multiple channels is challenging when cutting annular metal workpieces [13]. In addition to semiconductor processing, extensive research has also been conducted on multi-electrode discharge machining of metal workpieces. Kunieda and Muto [14] devised a dual-electrode discharge system, where two electrodes are connected in series with the workpiece for multi-channel electrical discharge machining (EDM), achieving a single-pulse material removal rate 1.30 times higher than that of a single electrode. Yu et al. [15] implemented multi-channel EDM with aerosol dielectric by combining multiple independent tubular electrodes in series with resistors, resulting in a 92% increase in the cutting rate compared to single-channel methods. Yang et al. [16] employed six mutually insulated and capacitively isolated tool electrodes for multi-channel discharge machining, resulting in a material removal rate 4.02 times higher than that achieved with a single electrode. In the above research, when multiple electrodes or workpieces were mutually insulated, it was possible to achieve multi-channel independent discharge and a certain level of improvement in the cutting rate. However, for the multi-channel HSWEDM of annular metal workpieces, the wire electrode and the workpiece are inseparable units, rendering it impossible to directly employ the aforementioned method for achieving multi-channel discharge and enhancing the cutting rate.

The pulse power supply provides pulse energy for WEDM, and its circuit topology has a significant impact on the distribution and utilization of discharge energy. Typically, the discharge characteristics can be improved and practical processing issues can be addressed by designing the power supply topology. In order to generate nanosecond pulse energy, Yan and Liu [17] proposed a fixed pulse-width modulation pulse control method and an anti-electrolysis circuit topology to improve the surface quality of tungsten carbide. Deng et al. [18] designed a high-low voltage composite circuit topology to enhance the processing efficiency of insulated ceramics. Kane et al. discussed a dual-converter-based topology and proposed a pulsed current reference scheme that improved energy efficiency from 31% to 62% [19]. In order to improve the dynamic response and power efficiency of the pulse power supply in EDM, Yang et al. designed an adaptive voltage position control power topology, achieving energy efficiency higher than 80% and high machining efficiency [20]. Therefore, the implementation of a specialized power supply design can significantly enhance the machining performance of WEDM, increase the cutting rate of metal workpieces, and provide a feasible approach for multi-channel discharge.

This paper examines the discharge characteristics in multi-channel HSWEDM utilizing a conventional power supply and reveals the reason why each channel cannot discharge independently. Building upon this analysis, a new power supply with decoupled circuits is proposed to enable synchronous discharges in multi-channel HSWEDM and enhance the cutting rate. The impact of current-limited resistance on synchronous discharges is thoroughly investigated. Experimental results validate the effectiveness of the new power supply in significantly improving the cutting rate.

## 2. Analysis of Multi-Channel HSWEDM Processing

### 2.1. Electrical Model Analysis of Four-Channel HSWEDM

**Figure 2:**
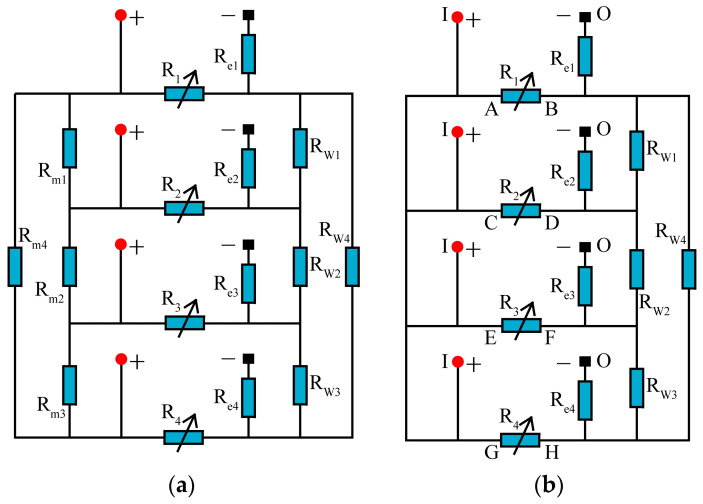
Equivalent circuit of four-channel HSWEDM: (**a**) equivalent circuit; (**b**) simplified equivalent circuit.

The equivalent circuit of the multi(four)-channel machine tool shown in Figure 1 is depicted in Figure 2a. *R* (*R*_1_–*R*_4_) represent the interelectrode gap resistances of channels 1–4, respectively. It should be noted that the resistance of *R*_1_–*R*_4_ is subject to variation depending on the gap environment [21]. When no discharge occurs and the gap remains unbroken, it can be considered an open circuit, which means that *R*_1_–*R*_4_ would be infinite. *R_m_* (*R_m_*_1_–*R_m_*_4_) represent the internal resistance of the annular metal workpiece, which can be considered negligible due to its low resistivity (0.73 × 10^−6^ Ω·m^2^/m). *R_e_* (*R_e_*_1_–*R_e_*_4_) represent the wire electrode resistances between the conductive block and the discharge point. *R_w_* (*R_w_*_1_–*R_w_*_4_) represent the resistance of the electrode wire between channels. The wire electrode has a large span between channels and a small diameter, resulting in resistances of *R_w_*_1_–*R_w_*_4_ ranging from 2 to 6 Ω, which cannot be neglected. Therefore, the equivalent circuit can be simplified, as depicted in Figure 2b.

In order to elucidate the coupling relationship between the gap voltage of each channel, a thorough analysis of the potential at each point in the circuit is conducted. By referring to Figure 2b, the potential relationship of Points *I*, *A*, *C*, *E*, and *G* can be determined.

(1) $$\varphi_{A}=\varphi_{C}=\varphi_{E}=\varphi_{G}=\varphi_{I}$$

Under the assumption that only the gap of channel 1 breaks down, an analysis of the gap voltage in the other three channels enables us to establish the relationship between the potential at Points *B*, *D*, *F*, and *H*.

(2) $$\left\{\begin{array}{c} U_{Rw1}=\varphi_{B}-\varphi_{D}\\ U_{Rw1}+U_{Rw2}=\varphi_{B}-\varphi_{F}\\ U_{Rw4}=\varphi_{B}-\varphi_{H} \end{array}\right.$$

By combining Equations (1) and (2), the relationships between the gap voltages can be obtained.

(3) $$\left\{\begin{array}{c} U_{CD}=U_{AB}-U_{Rw1}\\ U_{EF}=U_{AB}-\left(U_{Rw1}+U_{Rw2}\right)\\ U_{GH}=U_{AB}-U_{Rw4} \end{array}\right.$$

The currents passing through the resistance elements *R_e_*_1_–*R_e_*_4_ are identified as *I_Re_*_1_, *I_Re_*_2_, *I_Re_*_3_, and *I_Re_*_4_, respectively. Equation (4) can be derived by applying Ohm’s law and Kirchhoff’s law of current.

(4) $$\left\{\begin{array}{c} U_{Rw1}=R_{w1}\left(\begin{array}{c} \frac{R_{w2}+R_{w3}+R_{w4}}{R_{w1}+R_{w2}+R_{w3}+R_{w4}}\times I_{Re2}+\\ \frac{R_{w3}+R_{w4}}{R_{w1}+R_{w2}+R_{w3}+R_{w4}}\times I_{Re3}+\\ \frac{R_{w4}}{R_{w1}+R_{w2}+R_{w3}+R_{w4}}\times I_{Re4} \end{array}\right)\\ U_{Rw2}=R_{w2}\left(\begin{array}{c} \frac{R_{w3}+R_{w4}}{R_{w1}+R_{w2}+R_{w3}+R_{w4}}\times I_{Re3}+\\ \frac{R_{w4}}{R_{w1}+R_{w2}+R_{w3}+R_{w4}}\times I_{Re4}-\\ \frac{R_{w1}}{R_{w1}+R_{w2}+R_{w3}+R_{w4}}\times I_{Re2} \end{array}\right)\\ U_{Rw3}=R_{w3}\left(\begin{array}{c} \frac{R_{w4}}{R_{w1}+R_{w2}+R_{w3}+R_{w4}}\times I_{Re4}-\\ \frac{R_{w1}}{R_{w1}+R_{w2}+R_{w3}+R_{w4}}\times I_{Re2}-\\ \frac{R_{w1}+R_{w2}}{R_{w1}+R_{w2}+R_{w3}+R_{w4}}\times I_{Re3} \end{array}\right)\\ U_{Rw4}=R_{w4}\left(\begin{array}{c} \frac{R_{w1}}{R_{w1}+R_{w2}+R_{w3}+R_{w4}}\times I_{Re2}+\\ \frac{R_{w1}+R_{w2}}{R_{w1}+R_{w2}+R_{w3}+R_{w4}}\times I_{Re3}+\\ \frac{R_{w1}+R_{w2}+R_{w3}}{R_{w1}+R_{w2}+R_{w3}+R_{w4}}\times I_{Re4} \end{array}\right) \end{array}\right.$$

From Equation (4), we can write

(5) $$\left\{\begin{array}{c} U_{Rw1}=\frac{R_{w1}}{R_{T}}\left[\begin{array}{c} \left(R_{w2}+R_{w3}+R_{w4}\right)\times I_{Re2}+\\ \left(R_{w3}+R_{w4}\right)\times I_{Re3}+R_{w4}\times I_{Re4} \end{array}\right]\\ U_{Rw2}=\frac{R_{w2}}{R_{T}}\left[\begin{array}{c} \left(R_{w3}+R_{w4}\right)\times I_{Re3}+\\ R_{w4}\times I_{Re4}-R_{w1}\times I_{Re2} \end{array}\right]\\ U_{Rw3}=\frac{R_{w3}}{R_{T}}\left[\begin{array}{c} R_{w4}\times I_{Re4}-R_{w1}\times I_{Re2}-\\ \left(R_{w1}+R_{w2}\right)\times I_{Re3} \end{array}\right]\\ U_{Rw4}=\frac{R_{w4}}{R_{T}}\left[\begin{array}{c} R_{w1}\times I_{Re2}+\left(R_{w1}+R_{w2}\right)\times I_{Re3}+\\ \left(R_{w1}+R_{w2}+R_{w3}\right)\times I_{Re4} \end{array}\right] \end{array}\right.$$

where *R_T_* = *R_w_*_1_ + *R_w_*_2_ + *R_w_*_3_ + *R_w_*_4_. As the conductive block is near the processing position, *R_e_* << *R_w_*. Thus, *R_e_* can be ignored, so *I_Re_*_2_ ≈ *I_Re_*_3_ ≈ *I_Re_*_4_ ≈ 0. The relationship between the gap voltages of each channel can be represented by combining Equations (3) and (5).

(6) $$U_{CD}\approx U_{EF}\approx U_{GH}\approx U_{AB}$$

According to Equation (6), it can be inferred that the electrical signals of each channel in multi-channel HSWEDM exhibit a high degree of coupling. Once the voltage in one channel changes, the voltages in other channels will inevitably be altered accordingly.

### 2.2. Processing Analysis with a Conventional Power Supply

**Figure 3:**
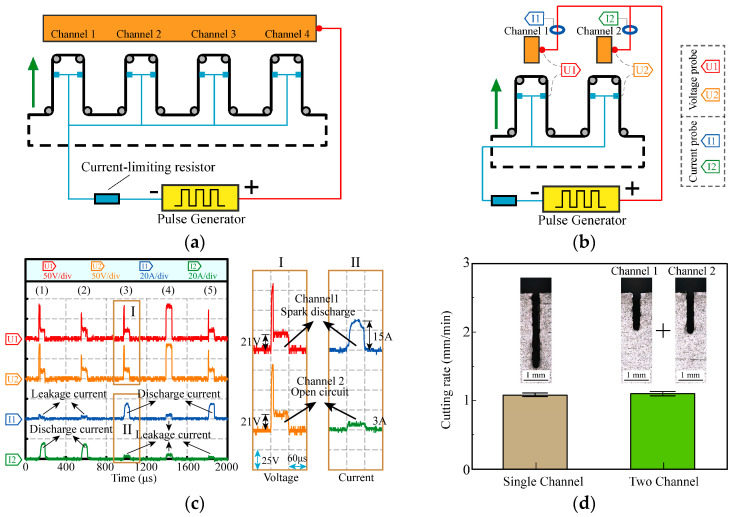
Processing by conventional power supply: (**a**) processing model of four-channel HSWEDM; (**b**) processing model of two-channel HSWEDM; (**c**) processing waveform; (**d**) cutting rate.

When using a conventional power supply to provide discharge energy, the processing model of four-channel HSWEDM is illustrated in Figure 3a. The power supply of HSWEDM typically comprises a pulse generator and a current-limiting resistor, with the positive pole connected to the workpiece and the negative pole connected to the wire electrode. To simplify the research, the processing model of two-channel HSWEDM is studied in this paper, as depicted in Figure 3b. In order to detect current waveforms, the workpiece is divided into two pieces and connected by copper wires. The detection positions of the probes are illustrated in Figure 3b. The voltage probe collects the gap voltage, while the current probe captures the discharge current.

Experiments were performed on single-channel and two-channel HSWEDM machine tools with a conventional power supply to investigate their processing characteristics. The workpiece material used was ASTM1045 steel (Anyang Iron and Steel Group Corporation; Anyang, China), and the processing parameters were set to an open voltage of 90 V, a current-limiting resistance of 5 Ω, a duty cycle of 1/7, a pulse width of 60 μs and a processing time of 3 min. Voltage and current waveforms were recorded during the process, and Figure 3c shows the processing waveforms in five consecutive pulse periods. It can be observed that in the five pulse periods, the voltage waveforms in the two channels are almost identical, while the current waveforms are different. Magnified views of the voltage and current in period 3 are shown in Parts I and II. The voltage of both channels drops rapidly to 21 V in Part I, exhibiting a typical spark discharge waveform [22,23]. However, in Part II, the corresponding current values for channels 1 and 2 in this pulse cycle were 15 A and 3 A, respectively. It is evident that the 15 A current in channel 1 is the discharge current, while the 3 A current in channel 2 is the leakage current. This indicates that spark discharge only occurs in channel 1, while there is no spark discharge in channel 2. During the WEDM process, discharge can take place only when the electric field strength in the gap meets the breakdown field strength of the working medium. Similarly, discharge exclusively takes place in channel 2 during period 1 and period 2. The current in period 4 is indicative of leakage current, implying an open circuit. Subsequently, discharge solely occurs in channel 1 during period 5. Thus, for each pulse period, the discharge occurs in one channel at most. Processing results are displayed in Figure 3d. Cutting rate is defined as the sum total of the feed speed in all machining channels. It can be observed that there are no significant variations in the cutting rate between single-channel and two-channel HSWEDM, maintaining an average of approximately 1.1 mm/min. This indicates that there is almost no improvement of the cutting rate in two-channel HSWEDM. Based on the analysis of processing waveforms, it can be seen that discharge does not occur simultaneously in the two channels within the same pulse period, limiting the improvement of cutting rate and not fully exploiting the advantages of multi-channel HSWEDM.

**Figure 4:**
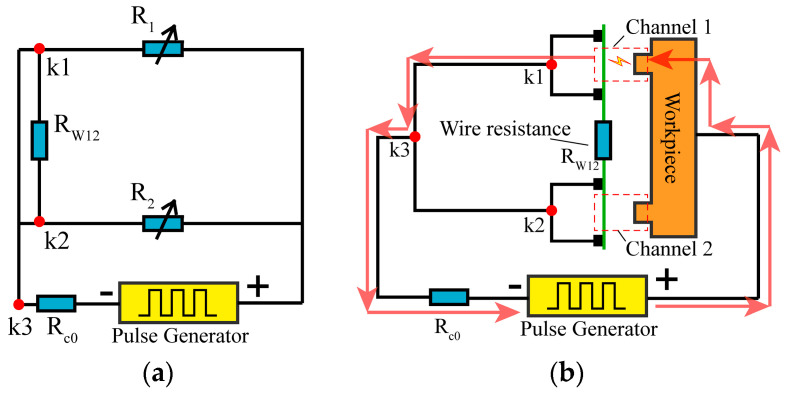
Multi-channel machining equivalent circuit diagram: (**a**) equivalent circuit; (**b**) discharge current loop with conventional power supply.

To further unveil the machining mechanism, an analysis of the circuit involved in the machining process was conducted. Figure 4a depicts the equivalent circuit for two-channel HSWEDM with a conventional power supply. Within this circuit, *R*_1_ and *R*_2_ represent the inter-electrode gap resistances of channel 1 and channel 2, respectively. *R_c_*_0_ represents the current-limiting resistance. *R_w_*_12_ denotes the equivalent resistance of the wire electrode between channel 1 and channel 2.

It can be observed that there are equipotential positive electrodes and negative electrodes between channel 1 and channel 2 (*U_R_*_1_ = *U_R_*_2_), thus the voltage waveforms in channel 1 and channel 2 are identical. In addition, as illustrated in Figure 4b, the detailed discharge process can be described as follows: when only the gap in channel 1 experiences breakdown, the current circuit follows the path indicated by the red arrows. The discharge current originates from the positive terminal of the power supply, passes through *k*_1_*k*_3_, and eventually returns to the negative terminal of the power supply. Meanwhile, the gap voltage in channel 1 decreases to the maintenance voltage. Since Points *k*_1_, *k*_3_, and *k*_2_ are equipotential points and the workpiece is also an equipotential body, the gap voltage in channel 2 is pulled down to the same maintenance voltage as in channel 1. The low level of the maintenance voltage hinders the breakdown of the gap in channel 2. Due to the coupling of electrical signals between the channels, each channel struggles to discharge independently, resulting in an inability to improve cutting speed.

### 2.3. The Design of the Decoupling Circuit for Multi-Channel HSWEDM

**Figure 5:**
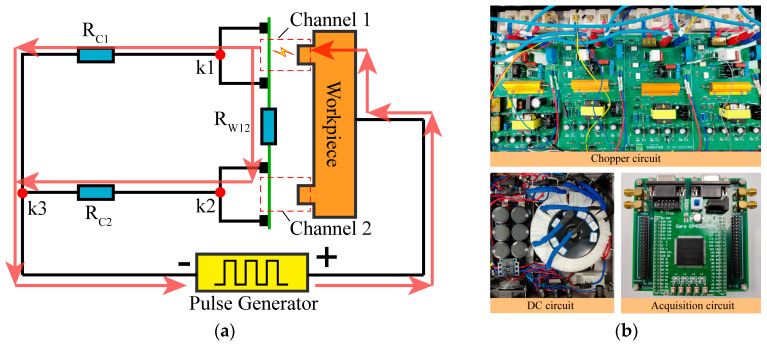
Design of MOP power supply: (**a**) discharge current loop with MOP power supply; (**b**) prototype of MOP power supply.

The equipotential between electrodes in different channels was discovered to be the basic cause of electrical signal coupling by the analysis in Section 2.2. It is clear that the effective way to accomplish synchronized discharge for multi-channel HSWEDM is to eliminate the equipotential between channels 1 and 2. Therefore, a Multi-Output Pulse (MOP) power supply with a decoupled circuit was designed for use in two-channel HSWEDM. The equivalent circuit is illustrated in Figure 5a, where the former *R_c_*_0_ from Figure 4b has been divided into *R_c_*_1_ and *R_c_*_2_ with identical resistance, connected to *k*_1_*k*_3_ and *k*_2_*k*_3_, respectively. When channel 1 discharges, a current loop is formed, as shown by the red arrow. The presence of *R_c_*_1_, *R_c_*_2_, and *R_w_*_12_ causes discrepancies in the potentials at *k*_1_ and *k*_2_, thereby weakening the electrical coupling between both channels. At this moment, the higher gap voltage provides beneficial discharge conditions for channel 2. The prototype diagram of an MOP power supply is shown in Figure 5b, including a chopper circuit, a DC circuit, and an acquisition circuit.

**Figure 6:**
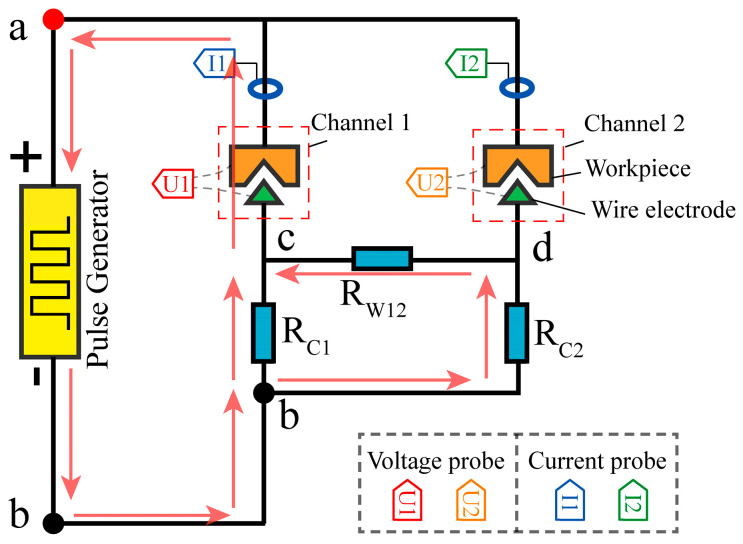
Two-channel discharge circuit diagram with MOP power supply.

To analyze the voltage relationship between the channels, we designed the discharge circuit with the MOP power supply as shown in Figure 6. The red arrow indicates the direction of the current flow. Points A, B, C, and D represent the electric potentials at key nodes in the circuit. When channel 1 experiences breakdown before channel 2, the relationship of voltage in different positions can be obtained according to Kirchhoff’s second law:

(7) $$\left\{\begin{array}{c} U_{ab}+U_{bc}+U_{ca}=0\\ {U_{cb}+U}_{bd}+U_{dc}=0\\ U_{ad}+U_{dc}+U_{ca}=0 \end{array}\right.$$

where *U_cd_* is the voltage drop corresponding to *R_w_*_12_, *U_cb_* is the voltage drop corresponding to *R_c_*_1_, *U_db_* is the voltage drop corresponding to *R_c_*_2_, *U_ab_* is the open voltage, *U_ac_* is the gap voltage of channel 1, and *U_ad_* is the gap voltage of channel 2.

In the stable discharge stage, the voltage relationship can be obtained from Ohm’s law as follows:

(8) $$\frac{U_{cd}}{U_{db}}=\frac{R_{w12}}{R_{c2}}$$

Combining Equations (7) and (8), the gap voltage of the non-breakdown channel (*U_ad_*) can be calculated as follows:

(9) $$U_{ad}=U_{ac}+\left(U_{ab}-U_{ac}\right)\times \frac{R_{w12}}{R_{w12}+R_{c2}}$$

where *R_c_*_1_ and *R_c_*_2_ are set to 5 Ω, and *U_ab_* = 90 V. When using a molybdenum wire with diameter of 0.18 mm, the actual measured resistance value of *R_w_*_12_ is 4 Ω.

After breakdown, the gap voltage rapidly drops from the open voltage to the maintenance voltage [24]. Based on previous experiments, a maintenance voltage of *U_ac_* = 21 V is selected. According to Equation (9), it can be determined that *U_ad_* = 51.7 V, which exceeds the maintenance voltage (*U_ac_*) in channel 1. Therefore, it is possible to induce the breakdown of the gap in channel 2.

**Figure 7:**
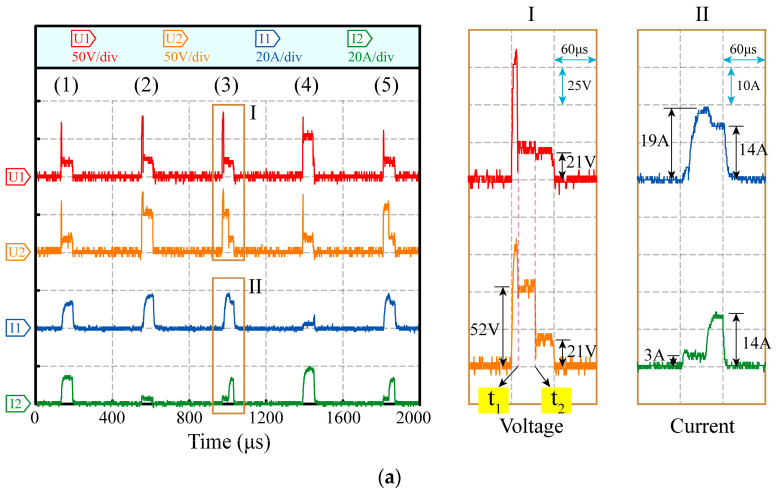

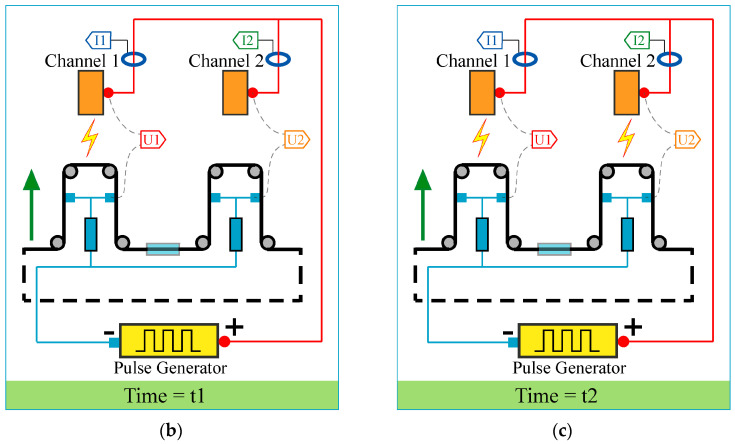
Voltage and current waveforms of two-channel processing with MOP power supply: (**a**) processing waveform; (**b**) discharge status at *t*_1_; (**c**) discharge status at *t*_2_.

The two-channel HSWEDM experiment was carried out using an MOP power supply to validate the aforementioned theoretical calculations. The processing parameters were configured as follows: an open circuit voltage of 90 V, a current-limiting resistance of 5 Ω, a duty cycle of 1/7 and a pulse width of 60 μs. The processing waveform is shown in Figure 7a, while Parts I and II display magnified views of the voltage and current, respectively, during period 3. In Figure 7b,c, the discharge status diagrams of Part I at times *t*_1_ and *t*_2_ are presented. The voltage waveforms in different channels exhibit differences, demonstrating the effectiveness of the decoupling circuit in mitigating the interaction between electrical signals in the two channels. Furthermore, based on the current waveforms observed during periods 1, 3, and 5, it is evident that there is a pronounced synchronous discharge phenomenon in both channels, with distinct variations in the current waveforms. Specifically, during period 3, the synchronous discharge process can be described as follows: When the gap of channel 1 breaks down at time *t*_1_, the maintenance voltage reduces rapidly to 21 V, and the discharge current reaches 19 A. Due to the decoupling circuit, the gap voltage of channel 2 maintains a high value at 52 V. Subsequently, when the gap of channel 2 breaks down at time *t*_2_, the discharge current rises from 3 A to 14 A. The process realizes the synchronous discharge at different channels in one pulse period. The theoretical calculation and experimental results prove that the MOP power supply can decouple the electrical signals in the two channels and realize synchronous discharge, thereby improving the cutting rate of multi-channel HSWEDM of metal workpieces.

In addition, Equation (9) indicates that the voltage division between *R_c_*_1_, *R_c_*_2_, and *R_w_*_12_ plays a crucial role in the decoupling process, which determines the division ratio of voltage. Since *R_w_*_12_ is a definite value, the study focuses on the impact of varying values of *R_c_*_1_ and *R_c_*_2_ on the gap voltage. When channel 1 undergoes breakdown before channel 2, rearranging Equation (9) yields

(10) $$\left\{\begin{array}{c} U_{ad}=U_{ac}+K\left(U_{ab}-U_{ac}\right)\\ K=\frac{R_{w12}}{R_{w12}+R_{c2}} \end{array}\right.$$

Because (*U_ab_* − *U_ac_*) > 0, U_ad_ increases as *K* increases. Since *R_w_*_12_ and *R_c_*_2_ are both greater than 0, and *R_w_*_12_ is 4 Ω, a smaller value of R_c2_ will result in a larger *K*. As *K* approaches 1, *U_ad_* approaches the open voltage *U_ab_*. In the following section, a detailed analysis will be conducted to study the impact of *R_c_*_1_ and *R_c_*_2_ on the multi-channel synchronous discharge, thereby confirming the enhancement of the cutting rate.

## 3. Experimental Procedures for Multi-Channel HSWEDM

**Figure 8:**
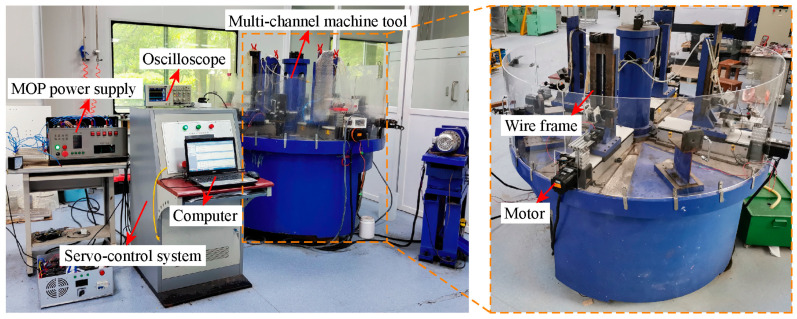
The schematic diagram of multi-channel machine tool.

Figure 8 presents the experimental setup of multi-channel HSWEDM. Each channel of the machine tool was equipped with a servo motor, allowing independent control of the feed rate for each channel based on feedback electrical signals. The discharge energy was provided by using either a conventional power supply or an MOP power supply. The power supply was equipped with a signal acquisition module that allows real-time collection of electrical signals during processing, which are fed back to the servo control system and computer. The computer recorded in real-time the various outputs generated by the experiment. The morphology was observed using a laser confocal microscope (OLS4100; Olympus, Japan). During the machining process, voltage and current signals were measured using a four-channel oscilloscope (MDO3104; Tektronix, Beaverton, OR, USA), a voltage probe (TPP0101; Tektronix, Beaverton, OR, USA), and a current probe (TCPA300; Tektronix, Beaverton, OR, USA). In this experiment, the workpiece consisted of multiple ASTM145 steel plates interconnected with copper wires. The specific experimental parameters are presented in Table 1.

## 4. Results and Discussion

### 4.1. Analysis of Gap Voltage

**Figure 9:**
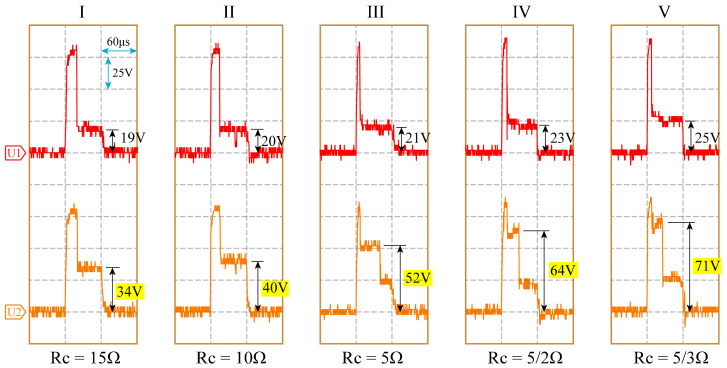
Processing waveforms of MOP power supply for different *R_c_*: (I) *Rc* = 15 Ω; (II) *Rc* = 10 Ω; (III) *Rc* = 5 Ω; (IV) *Rc* = 5/2 Ω; (V) *Rc* = 5/3 Ω.

Experiments were conducted under different *R_c_* values, with the experimental parameters presented in Table 1, where *R_c_*_1_ = *R_c_*_2_ = *R_c_*. Figure 9 displays the typical waveforms captured during processing with varying *R_c_* values. These waveforms clearly demonstrate that, following the initial breakdown in the gap of channel 1, the gap voltage in channel 2 remains at a relatively high level, measuring 34 V (15 Ω), 40 V (10 Ω), 52 V (5 Ω), 64 V (5/2 Ω), and 71 V (5/3 Ω), respectively. It can be observed that as the *R_c_* value decreases, the gap voltage in the non-breakdown channel increases, making the synchronous discharge process more pronounced.

**Figure 10:**
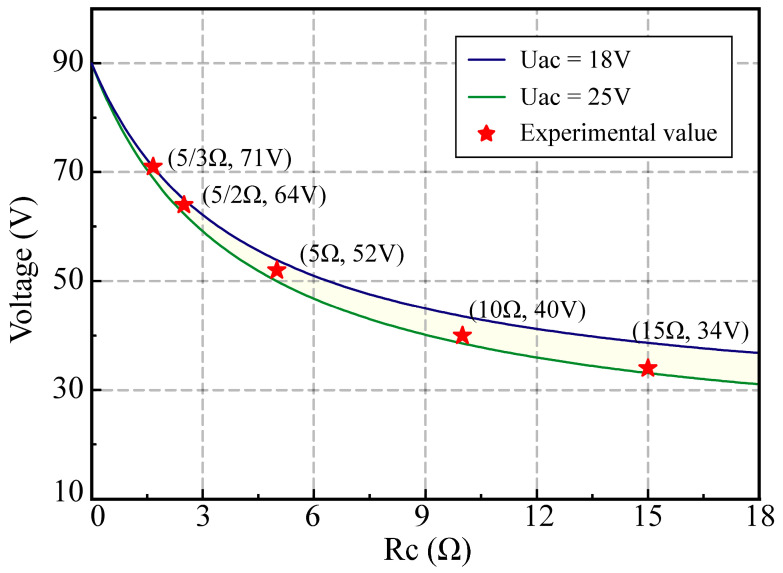
Theoretical values and experimental values of gap voltage for different *R_c_*.

Using Equation (9), the gap voltage of the non-breakdown channel can be calculated. Based on previous experimental data, the maintenance voltage after spark discharge typically falls within the range of 18–25 V, with the specific value dependent on the inter-electrode discharge status. Therefore, the gap voltage of the non-breakdown channel was calculated using sustaining voltages of 18 V and 25 V, resulting in two theoretical curves, as shown in Figure 10. The two theoretical curves enclose a theoretical region highlighted in yellow. The experimental values of the gap voltage (*U_ad_*) obtained from actual machining fall within the enclosed yellow region, indicating strong consistency between the theoretical and experimental values. Furthermore, as *R_c_* decreases and *K* increases, *U_ad_* approaches the open circuit voltage (*U_ab_*), suggesting a weakening coupling between the breakdown and non-breakdown channels. In WEDM, when other conditions are constant, a higher gap voltage results in a stronger electric field between the electrode wire and the workpiece. This increases the probability of reaching the breakdown field strength threshold of the dielectric fluid, which is advantageous for achieving synchronous discharge in multi-channel HSWEDM.

### 4.2. Analysis of Discharge Ratio

**Figure 11:**
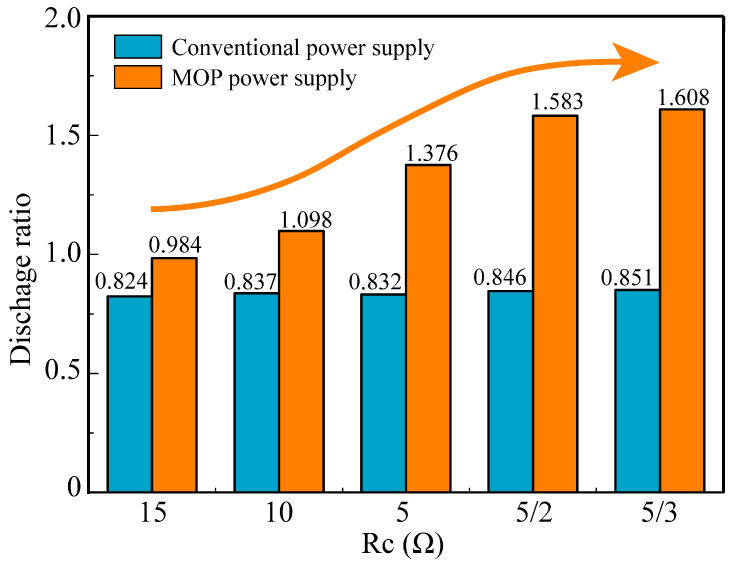
The discharge ratio of two-channel HSWEDM.

Experiments were conducted to investigate the discharge rate in multi-channel HSEDM using different *R_c_* values. The specific experimental parameters are presented in Table 1. Figure 11 illustrates the discharge ratio of two-channel HSWEDM under different *R_c_* values during continuous processing for 60 s. The discharge ratio is defined as the ratio of the number of discharges in all channels to the number of pulses emitted by the power supply. For the conventional power supply, the discharge ratio remains relatively constant at approximately 0.840, with little variation among different *R_c_* values. However, compared to the conventional power supply, the discharge ratio of the MOP power supply initially increases rapidly and then enters a relatively stable stage as *R_c_* decreases. Specifically, when *R_c_* decreases from 15 Ω to 5/2 Ω, the discharge ratio increases rapidly from 0.984 to 1.583. As *R_c_* further decreases from 5/2 Ω to 5/3 Ω, the discharge ratio gradually increases from 1.583 to 1.608. Combined with the analysis in Section 2.3, it can be inferred that as the value of *R_c_* decreases, the value of *K* increases, resulting in an increase in *U_ad_*, which in turn leads to a higher level of channel decoupling and a significant improvement in the discharge ratio. This observation highlights the ability of the MOP power supply to effectively decouple electrical signals between channels in multi-channel HSWEDM.

Based on Equation (10), when *R_c_* approaches 0 Ω, *K* approximates 1, resulting in *U_ad_* ≈ *U_ab_*. This implies that each channel of two-channel HSWEDM becomes completely independent, and at this point, the discharge rate and cutting speed of two-channel HSWEDM are theoretically twice that of single-channel HSWEDM. However, lower values of *R_c_* lead to excessively large currents, which in turn cause wire electrode breakage and diminish machining stability. This wire breakage phenomenon is observed in experiments with a *R_c_* value of 5/3 Ω. Therefore, in order to ensure stable machining, a moderate *R_c_* value of 5/2 Ω was chosen for subsequent cutting rate experiments.

### 4.3. Analysis of Cutting Rate

**Figure 12:**
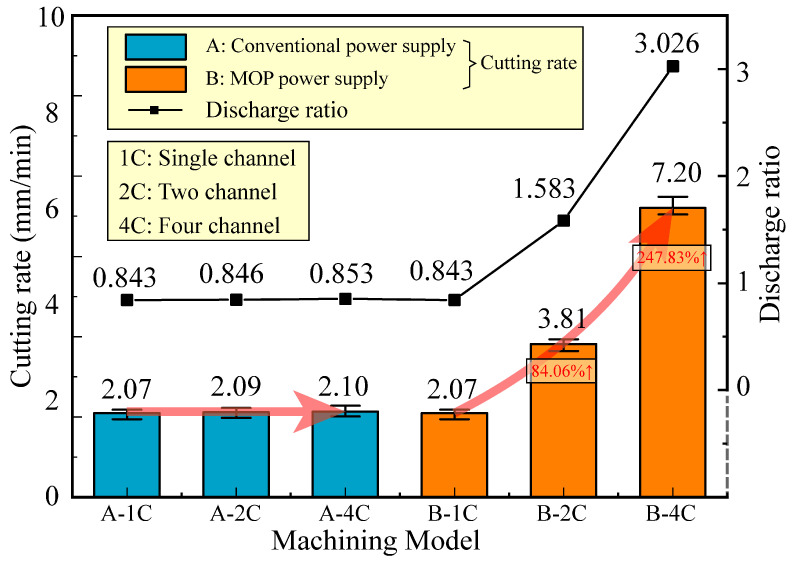
Processing state statistics with different machining models.

The experiments were carried out with *R_c_* = 5/2 Ω, and the other parameters are shown in Table 1. Figure 12 shows that the cutting rate and discharge ratio under different machining modes. When utilizing a conventional power supply, the discharge ratio remains relatively constant as the number of channels increases, staying at around 0.845. However, the use of the MOP power supply leads to a substantial increase in the discharge ratio with an increasing number of channels, escalating from 0.843 for single-channel HSWEDM to 3.026 for four-channel HSWEDM. The cutting rate demonstrates a strong correlation with the discharge ratio and exhibits a similar trend. In the cutting experiments using a conventional power supply, the cutting rates of single-channel, two-channel, and four-channel HSWEDM are nearly identical, ranging from 2.07 to 2.10 mm/min. On the other hand, using the new power supply, the cutting rate of single-channel HSWEDM is 2.07 mm/min. However, the cutting rates of two-channel and four-channel HSWEDM experience significant improvements, reaching 3.81 mm/min and 7.20 mm/min, respectively. This represents an increase of 84.06% and 247.83%, respectively. This improvement is primarily attributed to the MOP power supply reducing the degree of electrical signal coupling between channels, enabling synchronous discharge and enhancing the discharge ratio of multi-channel HSWEDM, thereby greatly improving the cutting rate.

**Figure 13:**
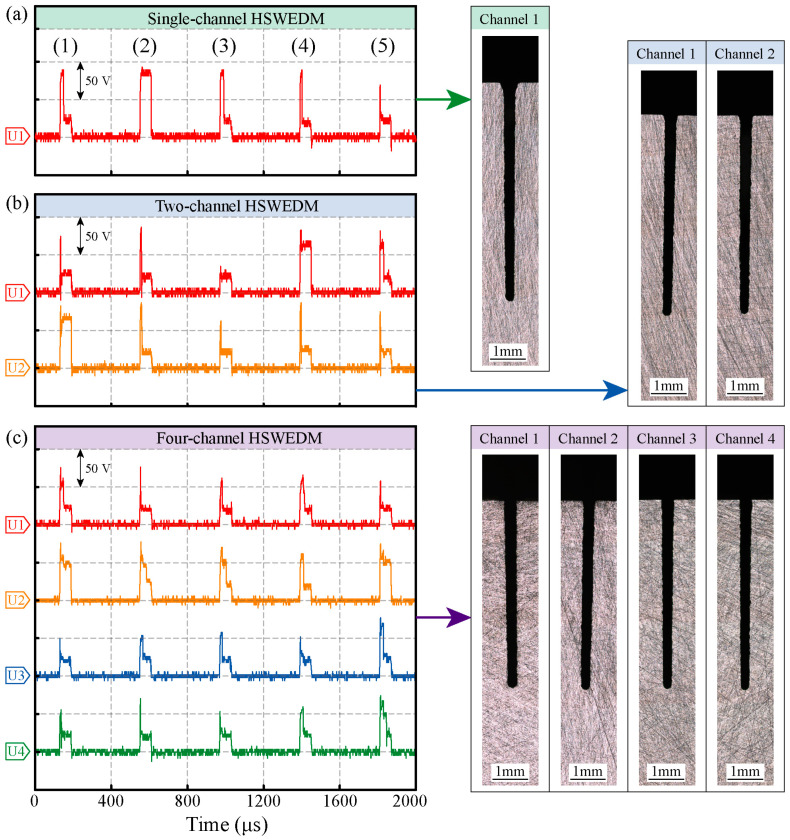
Processing results of MOP power supply. (**a**) single-channel HSWEDM; (**b**) two-channel HSWEDM; (**c**) four-channel HSWEDM.

The results of single-channel, two-channel, and four-channel HSWEDM using the MOP power supply are shown in Figure 13, including voltage waveforms and groove profiles. As the number of processing channels increases, it can be observed that the total groove depth significantly increases, indicating an improvement in cutting rate. From the voltage waveforms of two-channel and four-channel HSWEDM, it can be observed that there are significant differences in the voltage waveforms of each channel, indicating a substantial reduction in electrical signal coupling. In the voltage waveform for four-channel HSWEDM, during period 1, discharges occurred in channels 1, 3, and 4, while channel 2 remained in an open circuit state during this pulse stage. In period 2, discharges occurred successively in all four channels, with the discharge sequence being channel 4, channel 1, channel 3, and channel 2. This fully demonstrates that after decoupling with the MOP power supply, each channel can independently discharge. With the increase in discharge ratio, the cutting rate has also been significantly enhanced.

## 5. Conclusions

To address the issue of electrical signal coupling in multi-channel HSWEDM of a metal workpiece, an MOP power supply was designed to decouple the signals between channels, significantly enhancing cutting rate. The following conclusions are drawn:

1. A multi-channel HSWEDM machine tool was designed and manufactured, providing a realistic physical model for studying the multi-channel discharge mechanism in WEDM.
2. Through establishing the equivalent circuit, processing model, and synchronized acquisition of discharge waveforms for multi-channel HSWEDM, the study clarified that the equipotential between electrodes in different channels is the main factor responsible for electrical signal coupling between channels. This electrical signal coupling hampers multi-channel synchronous discharge, thereby posing challenges in enhancing cutting rate.
3. An MOP power supply was designed. By incorporating a specifically designed limiting resistor *R_c_*, the degree of electrical signal coupling between channels was significantly reduced. Smaller *R_c_* values resulted in a lower degree of coupling, a higher gap voltage, and a higher discharge ratio. In the case of two-channel HSWEDM, the discharge ratio increased from 0.984 at 15 Ω to 1.608 at 5/3 Ω.
4. The experimental results demonstrate that the use of an MOP power supply significantly enhances the processing performance of multi-channel HSWEDM. The discharge ratio increased from 0.843 in single-channel HSWEDM to 3.026 in four-channel HSWEDM. Compared to single-channel HSWEDM, the cutting rates of two-channel and four-channel HSWEDM are increased by 84.06% and 247.83%, respectively.

## Figures and Tables

**Table 1:** Experimental parameters of multi-channel HSWEDM.

Parameter	Value
Workpiece	ASTM1045 steel, cutting height of 30 mm
Diameter of tool electrode	Molybdenum wire, 0.18 mm
Working medium	BM-2 water-based fluid, conductivity of 3500 μS/cm
Power supply	MOP power supply/Conventional power supply
Open voltage	90 V
Pulse width	60 μs
Duty cycle	1/7
Current-limiting resistance	15 Ω,10 Ω, 5 Ω, 5/2 Ω, 5/3 Ω
Wire speed	8 m/s
Processing time	3 min

## Data Availability

Data are contained within the article.
